# Low-Dose Acetylsalicylic Acid Reduces T Cell Immune Activation: Potential Implications for HIV Prevention

**DOI:** 10.3389/fimmu.2021.778455

**Published:** 2021-11-18

**Authors:** Julie Lajoie, Monika M. Kowatsch, Lucy W. Mwangi, Geneviève Boily-Larouche, Julius Oyugi, Yufei Chen, Makobu Kimani, Emmanuel A. Ho, Joshua Kimani, Keith R. Fowke

**Affiliations:** ^1^ Laboratory of Viral Immunology, Department of Medical Microbiology and Infectious Diseases, University of Manitoba, Winnipeg, MB, Canada; ^2^ Department of Medical Microbiology, University of Nairobi, Nairobi, Kenya; ^3^ University of Nairobi Institute for Tropical and Infectious Diseases, University of Nairobi, Nairobi, Kenya; ^4^ College of Pharmacy, University of Manitoba, Winnipeg, MB, Canada; ^5^ Partners for Health and Development in Africa, Nairobi, Kenya; ^6^ Laboratory for Drug Delivery and Biomaterials, School of Pharmacy, University of Waterloo, Waterloo, ON, Canada; ^7^ Department of Community Health Science, University of Manitoba, Winnipeg, MB, Canada

**Keywords:** HIV, immune activation (IA), HIV risk, aspirin, Acetylsalicylic acid, inflammation, immune quiescence, HIV prevention

## Abstract

**Introduction:**

Acetylsalicylic acid (ASA) is a well-known and safe anti-inflammatory. At low-dose, it is prescribed to prevent secondary cardiovascular events in those with pre-existing conditions and to prevent preeclampsia. Little is known about how low-dose ASA affects the immune response. In this study, we followed women to assess how ASA use modifies T cells immune phenotypes in the blood and at the genital tract.

**Methods:**

HIV uninfected women from Kenya were enrolled in this study and followed for one month to assess baseline responses including systemic/mucosal baseline immune activation. Participants then received 81mg of ASA daily for 6 weeks to assess changes to T cell immune activation (systemic and mucosal) relative to baseline levels.

**Results:**

The concentration of ASA measured in the blood was 58% higher than the level measured at the female genital tract. In the blood, the level of ASA was inversely correlated with the following: the proportion of Th17 expressing HLA-DR (p=0.04), the proportion of effector CD4^+^ T cells expressing CCR5 (p=0.03) and the proportion of CD8^+^Tc17 expressing CCR5 (p=0.04). At the genital tract, ASA use correlated with a decreased of activated CD4^+^T cells [CD4^+^CCR5^+^CD161^+^ (p=0.02) and CD4^+^CCR5^+^CD95^+^ (p=0.001)].

**Conclusion:**

This study shows that ASA use impacts the immune response in both the systemic and genital tract compartments. This could have major implications for the prevention of infectious diseases such as HIV, in which the virus targets activated T cells to establish an infection. This could inform guidelines on ASA use in women.

**Clinical Trial Registration:**

ClinicalTrials.gov, identifier NCT02079077.

## Introduction

Acetylsalicylic acid (ASA) belongs to the non-steroidal anti-inflammatory drug (NSAID) category. Since its discovery, it has been used for the treatment of pain and fever (1). Currently, daily low dose ASA is prescribed for prevention of secondary cardiovascular events and stroke in patients with pre-existing conditions (2). Furthermore, USA guidelines were recently modified to include the use of low-dose ASA to reduce the risk of preeclampsia (3).

At low doses, ASA acts as an anticoagulation drug, which blocks the normal function of the cyclooxygenase (COX)-1 and -2 enzymes (4). By blocking the function of the COX enzymes, ASA prevents the synthesis of the pro-inflammatory lipids thromboxane (5) and some prostaglandins (6) while inducing the synthesis of the anti-inflammatory 15-epi-lipoxin (7). Furthermore, ASA inhibits the activation of NF-κB thereby blocking transcription of pro-inflammatory mediators (8) resulting in decreased infiltration of immune cells into tissues (9).

Immune activation and inflammation are risk factors for HIV infection. Recently, the CAPRISA 004 study, which compared a placebo control group to a 1% Tenofovir vaginal gel, showed the gel had significant protection from HIV infection (adjusted odds ratio 7.15 p=0.48) (10). However, it was also shown that regardless of the study arm they were in, participants with higher baseline of immune activation were more likely to acquire HIV (adjusted odds ratio 11.27 p=.009) where as an “innate immune quiescence” phenotype was protective (adjusted odds ratio 0.06 p=0.001) (10). Importantly, the presence of genital inflammation completely abrogated the protective effect of the Tenofovir gel demonstrating that inflammation can negate the protective effects of anti-viral agents (11). On the other hand, natural protection to HIV infection has been associated with an immune phenotype of reduced inflammation which our group has called “immune quiescence”. This phenotype is characterized by a lower baseline level in T cell activation and lower expression of pro-inflammatory chemokines (CXCL9 and CXCL10) (12). Together these studies show that genital inflammation impacts susceptibility to HIV infection and reducing inflammation is associated with protection from infection.

As inflammation is a major driver of disease progression among those infected with HIV, two clinical trials were conducted to determine if ASA could decrease inflammation in HIV-positive individuals. While one study showed ASA decreased the proportion of activated T cells (13), the second study showed no reduction in systemic inflammation or monocyte activation (14) suggesting that the tremendous immune activation caused by active HIV infection was not reversible with ASA. The impact of ASA use in the context of HIV infection remains debatable.

Until now, to our knowledge, no study looked at ASA to prevent HIV infection. Recently, we reported preliminary findings demonstrating that low-dose ASA treatment (81mg/day) decreased the proportion of HIV target cells at the female genital tract in HIV uninfected women and induced an immune quiescence phenotype similar to that observed in HIV-exposed seronegative (HESN) (15). Herein, we are reporting a detailed follow-up analysis to assess the immunological effects of how low-dose ASA altered the T cell compartment toward a less activated immune response which would have implications for a novel HIV prevention tool.

## Methods

### Study Design and Participants

As described previously, the Inducing Immune Quiescence (IIQ) study was a randomized pilot, open-label study conducted at the Pumwani maternity and Baba Dogo community clinics located in Nairobi, Kenya (15). Thirty-eight HIV uninfected non-sex worker women were randomized into the ASA arm and were given 81mg of ASA (Bayer Canada, Mississauga, ON) per day for six weeks. The second arm consisted of 39 women treated with 200 mg of hydroxychloroquine (HCQ). As the focus of this analysis is on the mechanism of the ASA effect, the HCQ arm will not be discussed in this paper (15). Briefly, enrolment criteria were age greater than 18 and less than 55 years, not self-declaring as sex workers, presence of the uterus and cervix, willing to take the study drugs for 6 weeks, in general good health, no recent pregnancy, not breastfeeding, not currently taking anti-inflammatory or immune-suppressors, being HIV uninfected and having no history of cardiovascular diseases. Exclusion criteria included being pregnant in the last 12 months, the presence of a sexual transmissible disease (STI) at enrolment or at any time during the course of the study, menopause, taking medication that counteracts with ASA, being allergic to the study drug, having history of heartburn, stomach pain, stomach ulcers, anomia, haemophilia, kidney or liver diseases, cardiovascular diseases, or being currently involved in another clinical trial. Both the University of Nairobi/Kenyatta National Hospital and the University of Manitoba research ethic boards approved this study. The study was registered on ClinicalTrials.gov (NCT02079077).

### Enrolment Procedures

Community consultative meetings were held prior to starting the study and served to introduce and discuss the study with community members, as well as assess their acceptance of the drugs, well before enrolment started. Eligible participants were screened and within two weeks were enrolled and randomized. All participants discussed the study with the study nurse and signed a consent form. Enrolled participants were followed for a 3-month period. As the immune system is known to vary during the menstrual cycle, vaginal sampling among women with a regular menstrual cycle was scheduled 5-8 days after the end of their menses. This study was designed without a placebo arm and each participant’s pre-drug baseline served as her own control. At visit 1 (pre-drug baseline) and visit 3 (final day of ASA, 6 weeks post initiation of drug) blood and cervical samples were taken for the assessment of systemic and mucosal immune activation. ASA was stopped at 6 weeks and an 8-week follow-up sample was collected to assess liver/kidney function and thromboxane levels.

### Sample Collection and Processing

Vaginal and blood samples were collected at each visit. A vulvovaginal swab was collected to assess for the presence of candida pseudohyphae and bacterial vaginosis by microscopy as well as *Trichomonas vaginalis*, which was diagnosed by In-Pouch kit (Biomed Diagnostics, USA). Urine samples were collected for detection of *Neisseria gonorrhea* and *Chlamydia trachomatis* (Roche Amplicor kits, USA). HIV serology using a rapid test (Determine, Inverness Medical, Japan) was performed at the first and last visit (there were no HIV seroconversions during or 6 months following the study). A questionnaire was completed at each visit. At the end of the study, to confirm adherence to the study protocol, plasma and cervico-vaginal lavage (CVL) were shipped to Winnipeg, Canada for ASA-level and cytokine and chemokine measurements (16).

Cervical cytobrushes and CVL were collected and processed according to the procedure detailed in Juno et al. (17). Blood was collected with venipuncture using heparin, and peripheral blood mononuclear cells (PBMC) were isolated by Ficoll density gradient.

### PBMC and CMC Flow Cytometry

Peripheral blood mononuclear cells (PBMC) (10^6^) and cervical mononuclear cells (CMC) were washed with 2% FBS-1x PBS and stained with PE.Cy5-CD3, FITC-CD4, V500-CD8, PE-CD95, APC.H7-HLA-DR, APC-CD161, Alexa700-CD45RA, V450-CCR5, PE.Cy7-CD69, PE-CF594-CCR7 (BD Biosciences, USA), or Far Red-Live Dead discriminant (Invitrogen, USA). Staining was done on fresh cells. Data were acquired on an LSRII flow cytometer (BD System, USA) and analysed using FlowJo v10.0.8r1 (TreeStar, USA). Gating strategy was to gate first on singlet, followed by lymphocytes gating and then gate on live dead and CD3^+^. Viable CD3^+^ cells were separated according to the CD4^+^ and CD8^+^ staining. For CMC, if fewer than 100 live cells were captured, the samples were excluded from the analyses. PBMCs not stained with the above panel were frozen and shipped to Winnipeg, Canada where two additional T cell phenotyping panels were used. For both assays, cells were thawed and rested overnight, 10^6^ PBMCs were washed with 2% FBS-1x PBS and for the first panel, targeted at T cell trafficking, stained with APC-fire-CD3, BV605-CD4, BV650-CD8, BV421-CD161, Alexa700-CD29, Pe-Dazzle-CXCR3, PerCP-Cy5.5-Vα7.2 (BD Biosciences, USA), PE-CD103, Pe-Cy7-CD25, BB515-CCR5, APC-CCR6 (Biolegend, USA), and Aqua Vivid Dead discriminant (ThermoFisher, USA). Cells were gated first on singlets, followed by lymphocytes and then on live dead. Live cells were gated on CD3^+^ and then separated into CD4^+^ and CD8^+^ subsets. Off the CD4^+^ group Th17 cells were gated as CD161^+^CCR6^+^. From the CD8^+^ group Tc17 cells were gated as CD161^+^Vα7.2-. MAIT cells were gated off the CD3 population then CD4- followed by CD161^++^Vα7.2^+^. All trafficking markers were then gated off each subset. For the second panel, for T regulatory (Treg) identification, cells were and stained following a modified intracellular (nuclear antigen) protocol from ThermoFisher Scientific (3). Antibodies used were as follows, PeCy7-CD3, BV605-CD4, BV650-CD8, Alexa488-CD25, Alexa700-CD127, Pe-Dazzle-TIGIT, APC-Fire-CD69, BV711-HLA-DR, PerCp-Cy5.5-CTLA-4 (Biolegend, USA), PE-FoxP3, eFlour450-Helios (eBiosciences, USA), and Aqua Vivid Dead discriminant (ThermoFisher, USA). Gating strategy consisted of gating on singlets followed by lymphocytes and then live cells. From there CD3^+^ cells were selected and then CD4^+^ cells. Tregs were defined off the CD4^+^ population as CD25^+^FoxP3^+^ and markers of activation and function were gated of the Treg population. To account for potential cytometer variability overtime 8 peak rainbow calibration particles (BD Biosciences, USA) were ran monthly and Fluorescent minus one controls were ran weekly using sample PBMCs. Median Fluorescence Intensity (MFI) of markers expressed on a per cell bases is described is utilized to describe “expression” of markers on a given cell.

### ASA Detection

ASA levels were compared between Visit 1 (baseline) and Visit 3 (6 weeks of ASA). ASA in plasma and CVL samples were quantitated using reversed phase high-performance liquid chromatography (RP-HPLC) following previously described methods (18) with slight modifications. Briefly, HPLC analysis was performed using a Symmetry^®^ C18 column (300Å, 3.5 µm 4.6 mm x 75 mm; Waters) with a Symmetry^®^ C18 guard column (300Å, 5 µm, 3.9 mm x 20 mm; Waters), fitted to a Waters^®^ Alliance^®^ HPLC system equipped with Waters^®^ 2690 Separations module and Waters^®^ 996 Photodiode Array detector. The mobile phase is comprised of water, acetonitrile and orthophosphoric acid at a ratio of 74:18:0.9 (v/v, pH 2.5). Each injection (100µL) was run for 10 min at 1 mL/min with a detection wavelength set at 234 nm. The retention time for the internal standard simvastatin (SIM) and ASA were 0.97 min and 5.09 min, respectively. Plasma samples (200µL) were combined with an equivalent volume of internal standard solution. The pH of the entire solution was adjusted to 2.7 by the addition of orthophosphoric acid and the analyte was extracted using 400 µL of acetonitrile. The supernatant was then transferred into a microcentrifuge tube containing 100-120 mg of sodium chloride, vortexed and centrifuged. The upper organic phase (100 µL) was injected for HPLC analysis. CVL samples were extracted similarly except the resulting supernatant was collected and evaporated under nitrogen gas. Following the addition of 100 µL of 0.01 M hydrochloric acid into the vial for reconstitution, the entire solution was injected into the HPLC machine. For ASA, the lower limits of quantitation (LLOQ) for CVL was 39 ng/mL and for plasma was 78 ng/mL with an extraction efficiency of 92.16 ± 0.64% for CVL and 86.45 ± 1.31% (mean ± SD) for plasma. A value of half the LLOQ was assigned to participants below the LLOQ, 19.5 ng/mL for CVL and 39 ng/mL for plasma.

### TXB2 Level

Thromboxane B_2_ (TXB2) levels were detected in the plasma using an enzyme-linked immunosorbent assay or ELISA at a 1:5 dilution as per the protocol provided by manufacturer (Abcam, Toronto, Canada) and ran on the Spectra Max Plus plate reader at 405nm with correction at 590 nm. Data was analysed using Wilcoxon matched-pairs signed rank on pg/mL raw data and visualized on a logarithmic scale for clearer visualization of the data.

### Cytokine and Chemokine Detection

Cytokines and chemokines were assessed using Milliplex MAP multiplex kits microbead assays (Millipore, Burlington, USA) and run using the Bio-Plex 200 (BioRad, Hercules, USA). Plasmas were incubated for 2 hours and room temperature and CVLs were incubated overnight at 4°C as per manufacturers protocol. Cytokine and chemokines and their lower limit of detection ran were as follows: 0.8 pg/mL for IFN-γ, 1.1pg/mL for IL-10, 0.6 pg/mL for IL-12(p70), 5.1 pg/mL for sCD40L, 0.7 pg/mL for IL-17A, 9.4 pg/mL for IL-1α, 0.8 pg/mL for IL-1β, 1 pg/mL for IL-2, 0.4 pg/mL for IL-8, 8.6 pg/mL for IP-10, 1.9 pg/mL for MCP-1, 2.9 pg/mL for MIP-1α, 3 pg/mL for MIP-1β, 0.7 pg/mL for TNF-α, 8.3 pg/mL for IL-1RA, 10.3 pg/mL for MIG, 1.6 pg/mL for MIP-3α and 6 pg/mL for IL-2RA. It should be noted that for samples below the limit of detection an assigned value of half the manufacturers specified limit of detection was used. For CVL sample 12 cytokines and chemokines were detected in enough abundance to be used in downstream analysis.

### Statistical Analysis

This was a pilot study to assess the impact of ASA on T cell immune activation. An intention to treat analysis was performed. Chi Squared (X^2^) was used to assess the significance of the associations between categorical variable using Prism 6.0f (GraphPad Software, La Jolla, CA, USA) and Gaussian distribution was tested by Shapiro-Wilk normality test and normality plot using SPSS (NY, USA). To compare baseline to visit 3 two-tailed paired T test (for normally distributed data) or Wilcoxon matched-pair signed-rank test (for data not following normality distribution) were performed using Prism 6.0f (GraphPad Software, La Jolla, CA, USA) or STATA v15.0 (StataCorp LLC, USA). Using SSPS, multivariate regressions were performed to assess the impact of DMPA usage and age on the change score (Visit 3-baseline). To understand the dynamics of the immune system between systemic and musical compartments we performed correlations between plasma and CVL ASA levels were run against data from both the PBMCs and CMCs. Pearson correlation or Spearman’s rank test were used for correlations between continuous variables (GraphPad Software, La Jolla, CA, USA). A p-value of <0.05 was considered as significant correction for multiple comparisons was not performed. Participants who were discontinued from the study were excluded from the analysis.

## Results

### Sociodemographics Information

**Table 1** describes the sociodemographic and clinical information about the participants. The majority of participants in this study practised vaginal douching, used depot medoxyprogesterone acetate (DMPA) as a family planning method and were in a relationship. Because age and use of DMPA are two factors known to modify the immune responses (19, 20), they were considered as confounders and entered in the multivariate regression model.

**Table 1:** Sociodemographics information about the participants in this study.

	ASA group n = 38
Age (mean (SD))	Range (20-45) 32 (8)
Practicing vaginal douching	21
Hormonal contraception	
No HC	12
Progesterone based	21
Oral pill	2
Other or not disclosed	3
BV status at visit 1	
Normal	20
Intermediate	14
Positive	4
Regular partner	
Yes	30
No	4
Not disclosed	4
TXB2 level (pg/ml) (mean (SD))	Range (32.4 – 27673)
Visit 1 (Baseline)	3279 (4314)
Visit 3	2287 (4740)
ASA level (ng/ml) at visit 3 (mean (SD))	Range (19.5 – 616)
Plasma	111.3 (134.7)
Cervico-vaginal lavage	46.14 (29.79)

n, number; SD, standard deviation; Range, minimum to maximum concentration detected in participant samples.

### TXB_2_ and Immune Response

**Figure 1:**
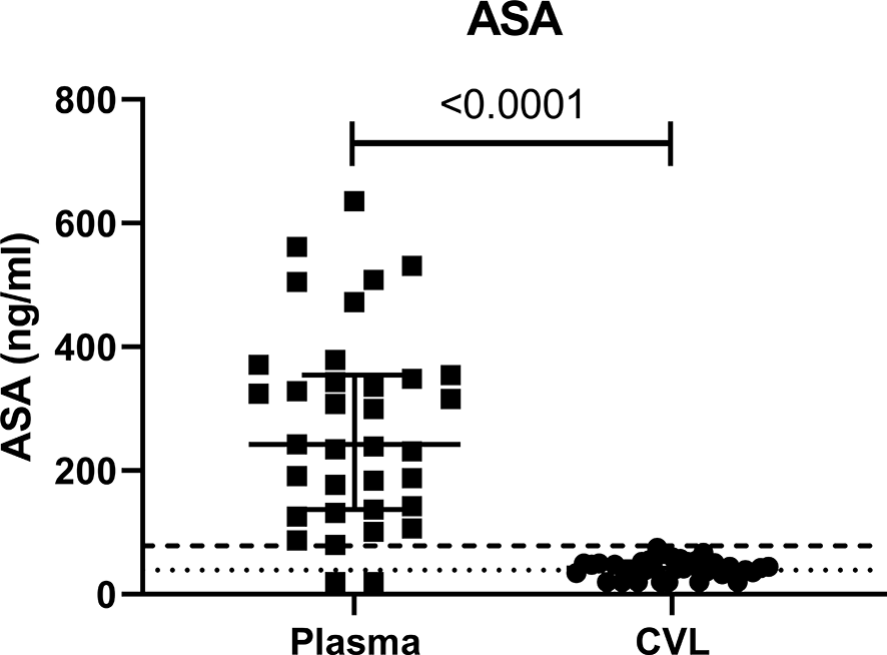
Concentration of ASA in ng/ml measured in the systemic (plasma) and female genital tract (CVL) compartment measured at visit 3 (six weeks after starting daily ASA 81mg regimen). CVL cervico-vaginal lavage. p values < 0.05 were considered significant. The lower limits of quantitation (LLOQ) for CVL was 39 ng/mL indicated by the dotted line and for plasma was 78 ng/mL indicated by the dashed line.

To ensure adherence to the study protocol, ASA concentration was measured at baseline and visit 3 (V3, six weeks after beginning of 81 mg ASA treatment). As suspected at baseline, we could not detect ASA in the blood or CVL of any of the participants (data not shown). At V3, ASA was detected in 94% of the participant blood and in 80% of the CVL. The concentration of ASA measured in the blood was 58% higher than the level measured at the female genital tract (**Figure 1**).

**Figure 2:**
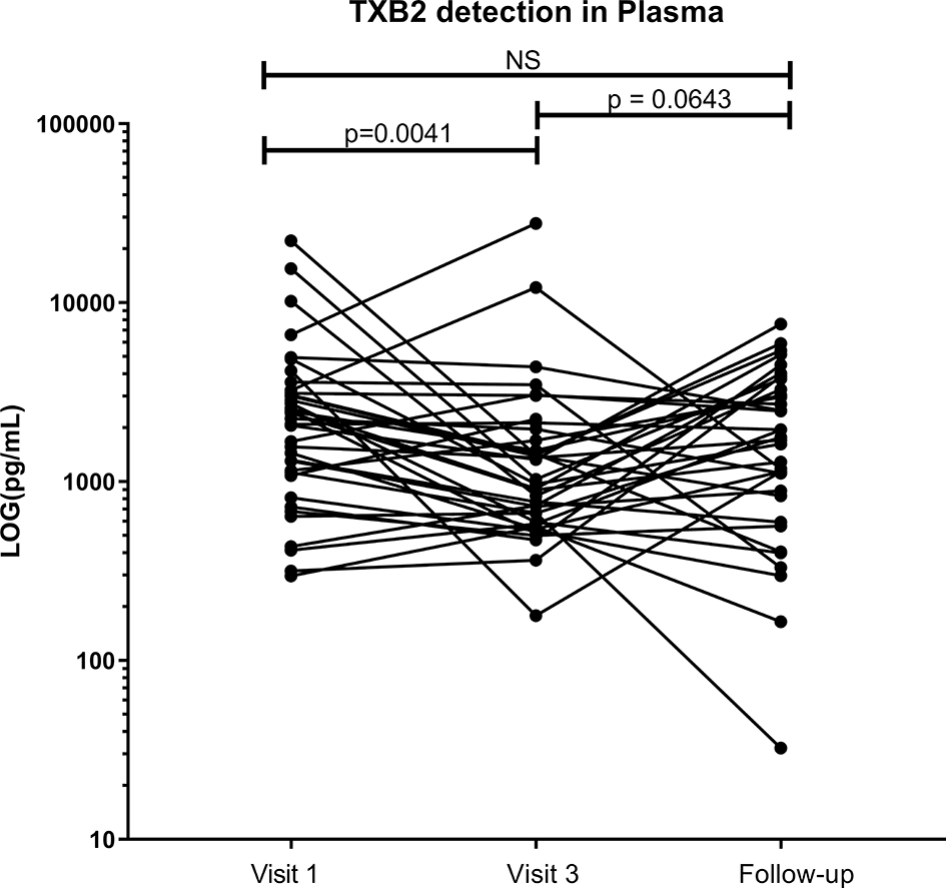
Effect of ASA uptake on TXB2 level between baseline (Visit 1), Visit 3 (after 6 weeks on 81mg ASA drug regimen) and at follow-up (two weeks after ending ASA regimen) in the systemic compartment (plasma). Data was analyzed using Wilcoxon matched-pairs signed rank test. p values < 0.05 were considered significant, NS, non-significant. Median [IQR] values for visit 1 and visit 3 can be found in **Supplementary Table S1**.

**Figure 3:**
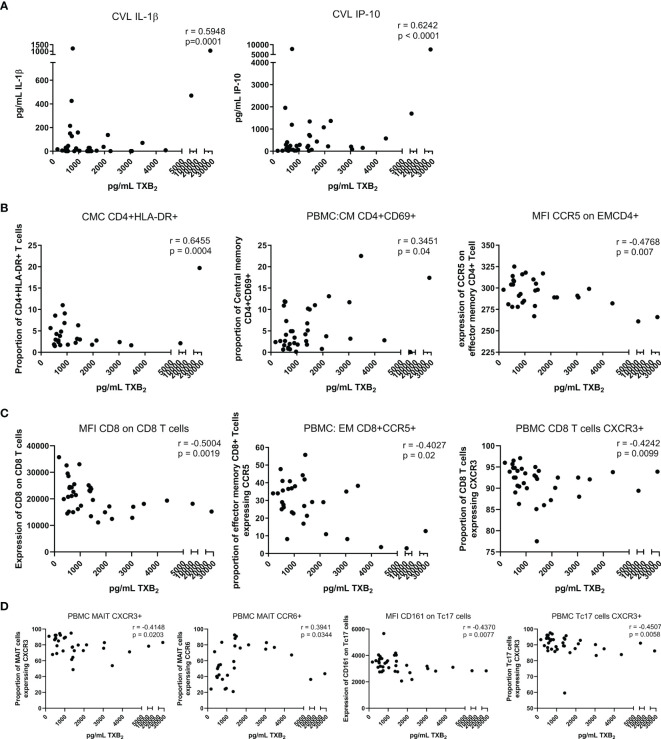
Correlation between the level of TXB2 in the plasma and immune activation at the mucosal and systemic compartment at visit 3 (six weeks after starting daily ASA 81mg regimen). **(A)** correlations between cytokines and chemokine and TXB2. **(B)** correlations between CD4^+^Tcell activation status and TXB2. **(C)** correlations between CD8^+^Tcell activation status and TXB2. **(D)** correlations between MAIT and Tc17 activation status and TXB2. Data was analyzed using Pearson correlation or Spearman’s rank test depending on normality of the data. p values<0.05 were considered significant. CVL, cervico-vaginal lavage; CMC, cervical mononuclear cells; PBMCs, peripheral blood mononuclear cells; CM, central memory cells; EM, effector memory cells; MFI, median fluorescent intensity indicates marker expression on a per cell bases.

The use of low-dose ASA correlated with a reduction in the formation of thromboxane B2 (TXB_2_). TXB_2_ is the metabolite of TXA_2_ a vasoconstrictor and a platelet coagulant (21) and therefore a direct measure of the pharmacological effect of ASA. TXB_2_ measurements allow determination of an individual responsiveness to ASA (22). In our study, the level of TXB_2_ dropped by 40% after six weeks on ASA, when compared to baseline level (p=0.004) and increased by 20% two weeks after the end of the treatment (**Figure 2**). The systemic level of TXB_2_ did not correlate with the concentration of ASA measured at the systemic and mucosal compartment (data not shown). At visit 3, the level of TXB_2_ did not correlate with cytokine/chemokine expression in the plasma but positively correlated with the level of IL-1β (p=0.0001) and IP-10 (p<0.0001) in the CVL (**Figure 3A**). TXB_2_ level also positively correlated with the mucosal proportion of CD4^+^HLA-DR^+^ T cells (p=0.0004). In the blood the proportion of central memory (p=0.04), effector memory CD4^+^ T cells (CD4^+^CD45RA^-^CCR7^-^) expressing CD69^+^ (p=0.0007) (**Figure 3B**) and the proportion of MAIT cells (CD4^-^CD161^++^Vα7.2^+^) expressing CCR6 (p=0.0344) (**Figure 3D**) positively correlated with the level of TXB_2_. In addition, the density of HIV entry co-receptor CCR5 on systemic effector memory CD4^+^T cells (p=0.007) (**Figure 3B**) and the proportion of MAIT cells expressing CXCR3 (p=0.0203) was inversely correlated with the TXB_2_ level (**Figure 3D**). Furthermore, the proportion of CD8^+^T cells expressing CXCR3 (p=0.0099) and proportion of effector memory CD8^+^ expressing CCR5 was inversely correlated with TXB_2_ (p=0.02) (**Figure 3C**). Finally, the expression of CD161 on Tc17 (CD8^+^CD161^+^) and the proportion of Tc17 cells expressing CXCR3 (p=0.0058) was inversely correlated with the level of plasma TXB_2_ (**Figure 3D**).

### Effect of ASA on Systemic Immune Activation in the Blood

**Figure 4:**
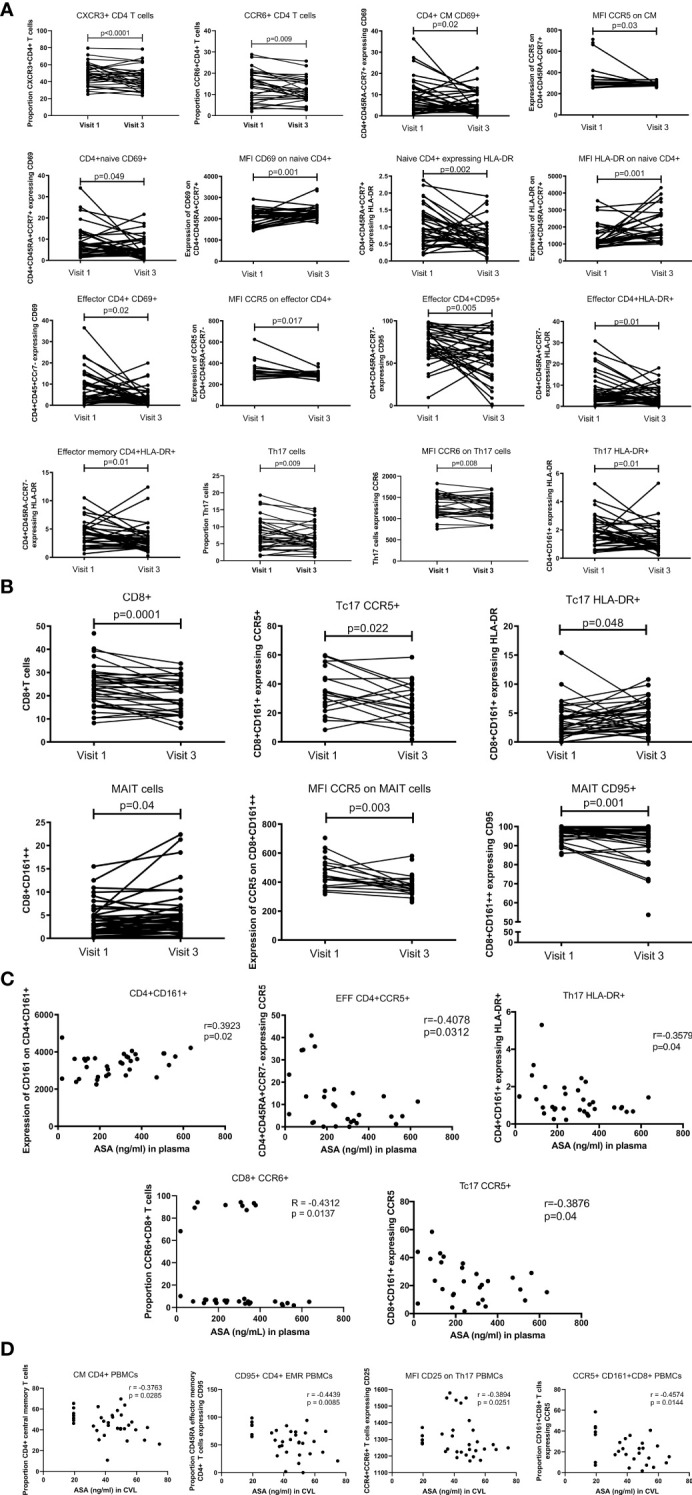
T cell blood proportion and immune activation status measured by flow cytometry. **(A)** systemic CD4^+^T cell activation changes between Visit 1 (baseline) and Visit 3 (after 6 weeks daily uptake of 81mg ASA). **(B)** systemic CD8^+^T cell activation changes between Visit 1 and Visit 3. **(C)** correlation between systemic T cell activation and blood concentration of ASA (ng/mL) at Visit 3. **(D)** correlation between systemic T cell activation and CVL concentration of ASA (ng/mL) at Visit 3. Data for **(A, B)** was analyzed using Wilcoxon matched-pairs signed rank test, multivariate regression was performed to control for the effect of DMPA and age on immune activation changes. Median [IQR] values for visit 1 and visit 3 can be found in **Supplementary Table S1**. Data for **(C, D)** was analyzed using Pearson correlation or Spearman’s rank test depending on normality of the data. p values<0.05 were considered significant. CM, central memory cells; MFI, median fluorescent intensity indicates marker expression on a per cell bases.

Using flow cytometry on frozen PBMCs, we assessed how low-dose ASA modifies T cell activation. The data presented herein are new and build on those data presented in our previous paper characterizing CD4^+^CCR5^+^ HIV target cells (15). After multivariate analysis, on bulk CD4 T cells from PBMCs, we observed a decrease in the proportion of CXCR3^+^CD4^+^T cells and CCR6^+^CD4^+^ (p<0.001 and p=0.009, respectively) (**Figure 4A**)

The proportion of central memory CD4^+^T cells (CD4^+^CCR7^+^CD45RA^-^) expressing CD69 (p=0.02) was also lower at V3 and the density of CCR5 expression was decreased on bulk central memory CD4^+^T cells (p=0.03). The proportion of naïve T cells (CD4^+^CD45RA^+^CCR7^+^) expressing the activation markers CD69 or HLA-DR was lower after six weeks of ASA regimen (p=0.049 and p=0.002 respectively). However, expression of these two markers increased on naïve CD4^+^T cells at V3 (p=0.01 and p=0.001 respectively), indicating that while there is less activation of CD4^+^ naïve T cells in the blood at V3 compared to the baseline each cell expressed higher quantity of CD69 or HLA-DR. Furthermore, we measured a lower proportion of activated effector CD4^+^ T cells (CD4^+^CD45RA^+^CCR7^-^) expressing the activation markers CD69, CD95 or HLA-DR after six weeks on ASA (p=0.02, p=0.005, p=0.01 respectively) as well as a decrease in the expression of CCR5 on bulk effector CD4^+^T cells (p=0.017). Finally, the proportion of effector memory T cells (CD4^+^CD45RA^-^CCR7^-^) expressing HLA-DR^+^ was decreased after ASA regimen (p=0.01) (**Figure 4A**). In our multivariate regression model, none of those associations were affected by the age or use of hormonal contraception (data not shown).

In addition, we also observed a decreased proportion of Th17 (defined as CD4^+^ CD161^+^) (p=0.009) and the proportion of Th17 expressing HLA-DR (CD4^+^CD161^+^HLA-DR^+^) (p=0.01) after use of ASA. The per cell intensity of expression of CCR6 on Th17 cells was also decreased (p=0.008) following 6 weeks on ASA (**Figure 4A**). The percent of Th17 cells positive for CD103 increased following 6 weeks ASA and the expression of inhibitory molecule TIGIT on T regs decreased following 6 weeks ASA in the univariate analysis (data not shown) although the changes of both populations did not remain significant in our multivariate model adjusting for age and method of contraception.

ASA use correlated with alterations in the CD8^+^ population in the blood. The proportion of CD8^+^T cells (p=0.0001) and Tc17 (defined as CD8^+^CD161^+^) expressing CCR5 (p=0.02) was lower at V3 compared to the baseline. The proportion of Tc17 expressing HLA-DR^+^ was increased after six weeks of ASA treatment (p=0.048). By measuring the expression of CD161 on CD8^+^T cells, we were able to assess the proportion and immune activation state of MAIT cells (defined as CD8^+^CD161^++^). MAIT cell proportion was increased at V3 (p=0.04), however, among the MAIT population the intensity of expression of CCR5 and the proportion of MAIT cells expressing CD95 were reduced (p=0.003 and p=0.001 respectively) (**Figure 4B**). Neither age nor use of DMPA affected the outcome observed following multivariate analysis.

When assessing at the concentration effect of ASA on the immune response, we observed that the expression of CD161 on CD4^+^T cells was positively correlated with the concentration of ASA measured in the blood(p=0.02). However, a negative correlation between the concentration of ASA was also observed with the proportion of effector CCR5^+^CD4^+^Tcells (p=0.03) and with the proportion of long-term activated HLA-DR^+^Th17 cells (p=0.04). The proportion of CCR5^+^Tc17 (p=0.04), and the proportion of CCR6^+^CD8 T cells (p=0.014) also exhibited a negative correlation with plasma ASA levels (**Figure 4C**). We also observed negative correlations between the CVL concentration of ASA and the proportion of CCR5^+^CD161^+^CD8^+^T cells (p=0.014), CD95^+^CD4^+^CD45RA^+^ effector memory T cells (p=0.008), the proportion of central memory CD4^+^T cells (p=0.029), and the expression of CD25 on Th17 cells (p=0.025) (**Figure 4D**).

### Effect of ASA on Mucosal Immune Activation in the FGT

**Figure 5:**
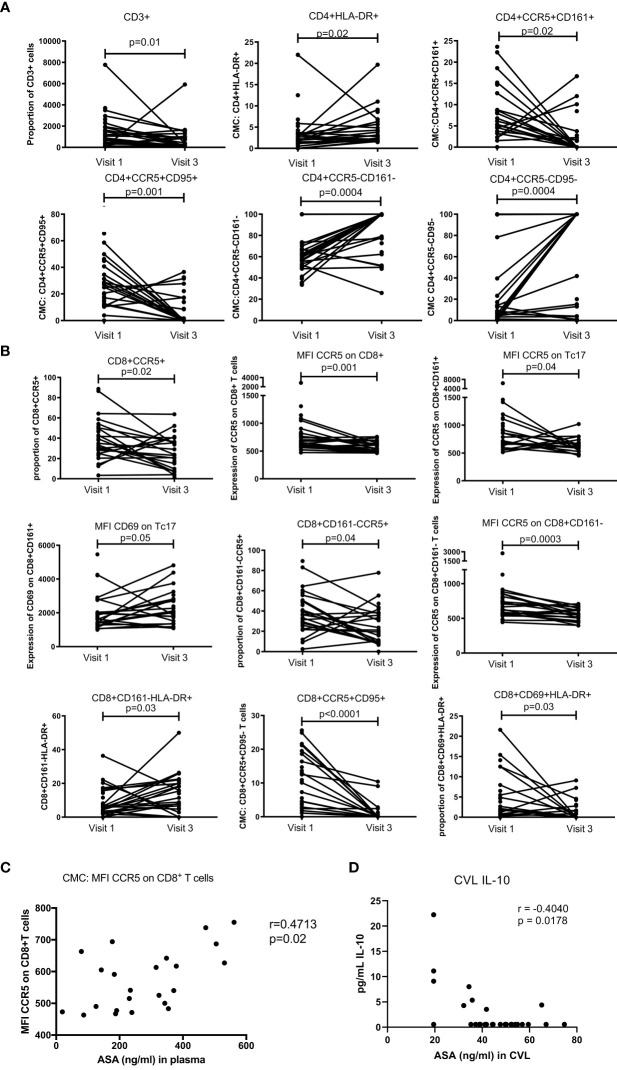
T cell mucosal proportion and immune activation status measured by flow cytometry on cervical mononuclear cells. **(A)** mucosal CD4^+^T cell activation changes between Visit 1 (baseline) and Visit 3 (after 6 weeks daily uptake of 81mg ASA). **(B)** mucosal CD8^+^T cell activation changes between Visit 1 and Visit 3. **(C)** correlation between mucosal T cell activation and blood concentration of ASA (ng/mL) at Visit 3. **(C)** correlation between mucosal T cell activation and CVL concentration of ASA (ng/mL) at Visit 3. Data for **(A, B)** was analyzed using Wilcoxon matched-pairs signed rank test, multivariate regression was performed to control for the effect of DMPA and age on immune activation changes. Median [IQR] values for visit 1 and visit 3 can be found in **Supplementary Table S1**. Data for **(C, D)** was analyzed using Pearson correlation or Spearman’s rank test depending on normality of the data. p values<0.05 were considered significant. MFI, median fluorescent intensity indicates marker expression on a per cell bases.

Fresh cervico-mononuclear cells were also analysed by flow cytometry. We observed that at the female genital tract compartment, use of ASA led to a decrease in the proportion of CD3^+^T cells (p=0.01). The proportion of CD4^+^HLA-DR^+^ increased between baseline and visit 3 (p=0.02). Activated double positive CD4^+^CCR5^+^CD161^+^ (p=0.02) and CD4^+^CCR5^+^CD95^+^ (p=0.001) decreased with ASA use while the proportion of the double negative CD4^+^CCR5^-^CD161^-^ (p=0.0004) and CD4^+^CCR5^-^CD95^-^ (p=0.0004) increased (**Figure 5A**). All these associations remained significant when controlled for age and DMPA use in the multivariate analysis (data not shown). CD4^+^CCR5^+^ HIV target cells were characterized in a previous paper (15).

For the CD8^+^T cell population, we observed a reduced level of CD8^+^CCR5^+^T cells (p=0.02). The expression of CCR5 on CD8^+^T cells and Tc17 was also decreased (p=0.001 and p=0.04 respectively). The density of CD69 on Tc17 was, however, increased from baseline to visit 3 (p=0.05). In the population of CD8^+^T cells not expressing CD161, the proportion of those carrying CCR5 was slightly decreased (p=0.04). Furthermore, the expression of CCR5 on those cells (CD8^+^CD161^-^) was also lower after ASA use (p=0.0003). The proportion of CD8^+^CD161^-^HLA-DR^+^ was increased between baseline and visit 3 (p=0.03). Finally, the proportion of double positive CD8^+^CCR5^+^CD95^+^ and CD8^+^CD69^+^HLA-DR^+^ was reduced between baseline and the end of ASA regimen (p=0.001 and p=0.03 respectively) (**Figure 5B**). None of those associations were affected by age or DMPA use.

Finally, the expression of CCR5 on CD8^+^T cells positively correlated with the concentration of ASA measured in the blood (p=0.02) (**Figure 5C**). The concentration of ASA measured in the CVL did not correlate with any T cell populations. However, we did observe a negative correlation between the CVL concentration of ASA and IL-10 (p=0.018) (**Figure 5D**).

## Discussion

ASA is readily available worldwide. It has been used for decades for the treatment of pain and in more recent years to prevent heart diseases as well as pre-eclampsia (2, 21). In this study, we assessed how ASA use modifies the T cell immune response. We observed that six weeks of ASA treatment decreased T cell immune activation in HIV negative women.

Previous studies showed that about 50-70% of the initial dose of ASA passes into the systemic circulation (23). In our study, we observed that ASA can also pass the mucosal barrier and be detected at the female genital tract. To our knowledge, this is the first study showing that ASA can be measured in vaginal secretion. We also measured the level of TXB_2_, which is inhibited by ASA and a direct measure of ASA activity. Herein, we observed a significant decrease in the level of TXB_2_ after six weeks of ASA use. The level of ASA and TXB_2_ measured indicates that the participants were highly adherent to the protocol and responded to the treatment.

In this study, we build upon our observations in HIV target cells (15) and observed that the proportion of chronically activated CD4^+^T cells (CD4^+^HLA-DR^+^) was inversely correlated with the level of ASA measured in the blood. This was also the case for the relation between ASA level and the proportion of effector CD4^+^T cells and Tc17 expressing HIV coreceptor CCR5. We previously reported that the mucosal level of ASA negatively correlated with the proportion of HIV target cells at the genital tract (15) and expand on our previous findings here demonstrating that the mucosal level of ASA negatively correlates with the proportion of CD161^+^CD8^+^T cells positive for CCR5 and the expression of activation marker CD25 on Th17 cells in the blood (24). Together, these results indicate that an increase concentration of ASA correlates with reduced inflammatory immune markers both in the blood and in the genital compartment.

It has been previously shown that ASA can impair T cell tissue recruitment by disrupting the integrin- and L-selectin-mediated binding of the T cells to the endothelium (25). In this study, we observed a lower proportion of CD3^+^ lymphocytes at the genital tract after ASA regimen compared to the baseline. We also observed a lower proportion of systemic central memory, naïve and effector CD4^+^T cells expressing CD69 as well as lower proportion of mucosal CD8^+^CD69^+^HLA-DR^+^T cells during ASA treatment. CD69 is an acute activation marker and a marker of resident T cells (26). The reduced proportion of T cells expressing CD69 may indicate that there are more circulating T cells and a lower proportion of those T cells remain resident in the genital tract. In addition to lower levels of CD69, we found that following ASA treatment the proportion of CD4^+^T cells expressing trafficking markers CCR6 and CXCR3 decreased as well as a decrease in the expression of CCR6 on Th17 cells. A decrease in CCR6 suggests ASA reduces the migration of these cells to mucosal tissues such as the intestine and colon (27), whereas decreases in CXCR3 indicate a reduction in lung and lymphoid tissue trafficking (28). We also found the concentration of ASA or TXB_2_ in the blood had a predominantly inverse relationship with these same trafficking markers on several T cell subsets. Together, our data corroborates with the study conducted by Gerli et al. (25) and suggest ASA affects lymphocytes trafficking.

T lymphocytes play a major role in the regulation of the adaptive immune response against pathogens. In the context of HIV infection, they are the main target cells for the virus. It has been shown that HIV can infect activated T cells 1000 times more efficiently than quiescent T cells (29). While on bulk CD4 T cells we observed an increase in the proportion of cells positive for HLA-DR at the female genital tract, on HIV target cells at the female genital tract the proportion of activated T cells CD4^+^CCR5^+^CD161^+^ and CD4^+^CCR5^+^CD95^+^ reduced with ASA. treatment Decreasing baseline immune activation in high-risk individuals could help increase the efficiency of other biomedical preventive methods such as microbicides affected by local inflammation as seen in the CARPRISA004 study.

Limitations of this study include the difficulty of recovering a sufficient number of CMCs for robust analyses for all markers tested. Other limitations include the limited sample size of 38 participants, only 6 weeks on therapy, and a single dose of ASA tested. As this was a discovery and hypothesis generating study, we did not perform corrections for multiple comparisons. To confirm these initial findings, follow up studies should be performed which narrow the focus of cytokine analysis to only a core set of cytokines thereby minimizing the comparisons to only those factors that would be hypothesized to be altered. Strengths of this study include paired systemic and mucosal sample analysis, in depth phenotypic characterization of multiple immune phenotypes, and the ability to correlate findings with tissue and systemic drug levels.

This study offers new insights into the immunological effects of how ASA affects inflammation. It also suggests a new approach in preventing HIV infection that could be combined with other prevention approaches to provide a wider selection of HIV prevention tools (29). We showed that ASA decreases T cell immune activation both at the systemic and female genital tract compartment. This is the first study that looks into how ASA impacts the immune response at the genital tract immune environment. In the context of HIV, this study shows that it is possible to modify to the female genital tract environment toward a less pro-inflammatory more immune quiescent milieu thereby potentially reducing HIV risk.

## Data Availability Statement

The original contributions presented in the study are included in the article/**Supplementary Material**. Further inquiries can be directed to the corresponding author.

## Ethics Statement

The studies involving human participants were reviewed and approved by the University of Nairobi/Kenyatta National Hospital and the University of Manitoba research ethic boards approved this study. The study was registered on ClinicalTrials.gov (NCT02079077). The patients/participants provided their written informed consent to participate in this study.

## Author Contributions

All authors participated in interpretation of data and critical review of the manuscript. JL participated in the study design, was the co-study coordinator, and wrote part of the article. MMK conducted the *in vitro* experiments and wrote part of the article. LM conducted many of the *ex vivo* experiments. GB-L was the field study coordinator and performed technical analyses. JO provided overall supervision of the Nairobi-based study. CY and EH performed ASA tissue concentrations. MK was the clinical officer and collected clinical samples. JK managed the clinical cohort. KF was the principal investigator of the study, obtained funding for the study and wrote the article.

## Funding

Funding support was provided by the Canadian Institutes of Health Research (CIHR) OCH # 126275 (KF, JL), CIHR HB3-164065 (KF, JL, JO, JK), Grand Challenge Canada S5 386-01 (JL) and CIHR PJT166153 (EH).

## Conflict of Interest

The authors declare that the research was conducted in the absence of any commercial or financial relationships that could be construed as a potential conflict of interest.

## Publisher’s Note

All claims expressed in this article are solely those of the authors and do not necessarily represent those of their affiliated organizations, or those of the publisher, the editors and the reviewers. Any product that may be evaluated in this article, or claim that may be made by its manufacturer, is not guaranteed or endorsed by the publisher.
