# Supplementary material for: Low-Dose Acetylsalicylic Acid Reduces T Cell Immune Activation: Potential Implications for HIV Prevention
Source: Front Immunol. 2021 Nov 18;12:778455. doi: 10.3389/fimmu.2021.778455 (PMC8637415; doi:10.3389/fimmu.2021.778455)
Supplement: Supplementary file 1 [file DataSheet_1.pdf]

1 SUPPLEMENTARY TABLES

Table S1. Median [IQR] values for visit 1 and visit 3 data from Wilcoxon-paired sign rank tests found in figures: 2, 4A, 4B, 5A, and 5B.

Figure number	Graph in question	Visit 1	Visit 3	Follow-up
Figure 2	TXB2 detection in Plasma	2245 [1101-3176]	969 [586-1829]	1736 [771-3390]
Figure 4A	CXCR3+ CD4 T cells	48.3 [40.1-59.2]	42.3 [33.6-50.2]	
	CCR6+ CD4 T cells	14.0 [6.69-19.1]	10.9 [8.15-16.3]	
	CD4+ CM CD69+	8.02 [2.92-12.50]	3.42 [1.79-10.10]	
	MFI CCR5 on CM	305.5[284.0-366.0]	283.0 [273.0-291.0]	
	CD4+naïve CD69+	7.38 [3.91-10.06]	4.16 [2.44-9.08]	
	MFI CD69 on naïve CD4+	2095 [1611-2290]	2202 [2119-2368]	
	Naïve CD4+ expressing HLA-DR	0.89 [0.53-1.36]	0.61 [0.35-0.89]	
	MFI HLA-DR on naïve CD4+	1061 [859-1642]	1581 [1148-2702]	
	Effector CD4+CD69+	5.26 [2.83-11.13]	2.76 [1.31-5.36]	
	MFI CCR5 on effector CD4+	315.5 [278.0-387.8]	288.0 [274.5-306.0]	
	Effector CD4+CD95+	72.80 [63.00-86.65]	59.60 [36.85-75.30]	
	Effector CD4+HLA-DR+	4.98 [3.44-9.04]	3.51 [1.47-7.74]	
	Effector memory CD4+HLA-DR+	4.17 [2.68-5.22]	2.85 [1.77-3.80]	
	Th17 cells	6.75 [3.96-10.5]	5.88 [4.00-8.09]	
	MFI CCR6 on Th17 cells	1290 [1092-1562]	1248 [1069-1457]	
	Th17 HLA-DR+	1.60 [0.94-2.34]	1.04 [0.76-1.87]	
Figure 4B	CD8+	24.40 [18.05-28.88]	21.70 [13.80-25.90]	
	Tc17 CCR5+	34.50 [24.40-44.10]	23.20 [13.20-35.80]	
	Tc17 HLA-DR+	3.42 [1.51-5.09]	4.39 [2.12-10.80]	
	MAIT cells	3.05 [1.81-5.81]	3.98 [2.145-6.54]	
	MFI CCR5 on MAIT cells	427.0 [397.8-512.0]	353.5 [321.0-408.0]	
	MAIT CD95+	97.65 [93.63-99.20]	95.20 [89.75-98.05]	
Figure 5A	CD3+	1106 [365-2290]	387 [140-990]	
	CD4+HLA-DR+	2.73 [1.53-3.47]	2.87 [1.83-5.82]	
	CD4+CCR5+CD161+	5.74 [2.99-13.20]	0.00 [0.00-3.02]	
	CD4+CCR5+CD95+	22.70 [11.63-36.05]	0.00 [0.00-17.05]	

	CD4+CCR5-CD161-	59.30 [49.50-70.00]	99.85 [63.80-100.0]	
	CD4+CCR5-CD95-	6.74 [2.15-17.30]	100.0 [4.15-100.0]	
Figure 5B	CD8+CCR5+	38.70 [27.48-52.30]	22.00 [7.54-37.38]	
	MFI CCR5+ on CD8+	698.0 [590.8-826.0]	546.5 [488.3-643.0]	
	MFI CCR5+ on Tc17	862.0 [665.0-1070.0]	623.5 [545.5-678.0]	
	MFI CD69 on Tc17	1627 [1287-1994]	2057 [1571-2827]	
	CD8+CD161-CCR5+	36.45 [24.30-50.65]	18.55 [8.74-37.75]	
	MFI CCR5 on CD8+CD161-	685.0 [563.5-822.3]	547.0 [464.0-639.0]	
	CD8+CD161-HLA-DR+	5.78 [3.51-14.30]	14.70 [6.32-20.28]	
	CD8+CCR5+CD95+	6.42 [1.61-15.13]	0.00 [0.00-0.84]	
	CD8+CD69+HLA-DR+	1.51 [0.32-5.49]	0.00 [0.00-1.97]	

2 SUPPLEMENTARY FIGURES

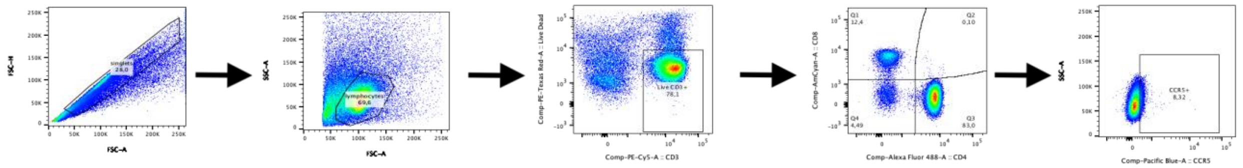

Figure S1. Gating strategy for *ex vivo* analysis of PBMCs and CMCs performed on fresh samples in Nairobi Kenya. CCR5 is provided as an example but other markers were analysed same way. Fluorescent minus one controls were performed weekly and 8-peak rainbow beads were performed monthly to standardize cytometer runs.

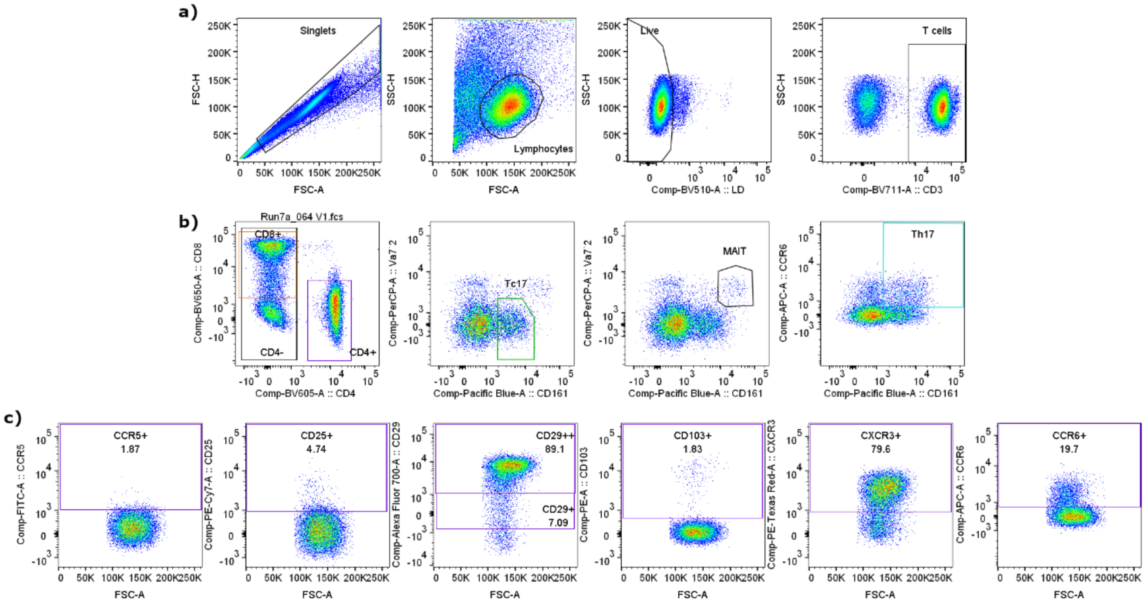

Figure S2. Gating strategy for *ex vivo* analysis of PBMCs performed on frozen samples from Nairobi Kenya. (A) outlines the strategy used for detection of CD3+T cells. (B) outlines the types

of T cells analysed in this panel, Tc17 cells were gated off CD8+ gate, MAIT cells were gated off CD4- gate, and Th17 cells were gated off CD4+ Gate. (C) outlines the markers used to characterize the aforementioned cell types. Fluorescent minus one controls were performed with every run and 8-peak rainbow beads were performed monthly to standardize cytometer runs.

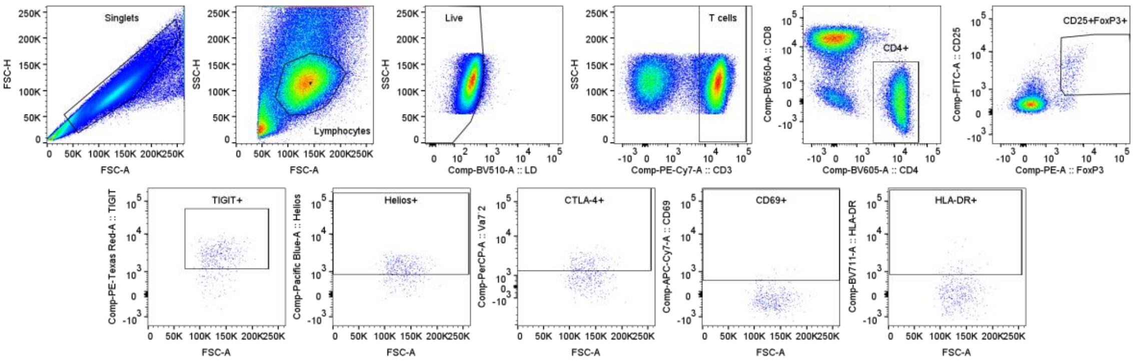

Figure 3S. Gating strategy for *ex vivo* analysis of PBMCs performed on frozen samples from Nairobi Kenya. Fluorescent minus one controls were performed weekly and 8-peak rainbow beads were performed monthly to standardize cytometer runs.
